# Spatiotemporal Distribution and Assemblages of Fishes below the Lowermost Dam in Protected Reach in the Yangtze River Main Stream: Implications for River Management

**DOI:** 10.1155/2016/4290793

**Published:** 2016-10-24

**Authors:** Junyi Li, Hui Zhang, Danqing Lin, Jinming Wu, Chengyou Wang, Xuan Xie, Qiwei Wei

**Affiliations:** ^1^College of Life Science, Southwest University, Chongqing, China; ^2^Key Laboratory of Freshwater Biodiversity Conservation, Ministry of Agriculture of China, Yangtze River Fisheries Research Institute, Chinese Academy of Fishery Sciences, Wuhan, China

## Abstract

Now more and more ecologists concern about the impacts of dam construction on fish. However, studies of fishes downstream Gezhouba Dam were rarely reported except Chinese sturgeon (*Acipenser sinensis* Gray). In this study, catch investigations and five hydroacoustic detections were completed from 2015 to 2016 to understand the distribution, size, and categories of fishes and their relationship with the environmental factors below Gezhouba Dam in protected reach in the Yangtze River main stream. Results showed significant differences in fish distribution and TS (target strength) between wet and flood seasons. Mean TS in five hydroacoustic detections were −59.98 dB, −54.70 dB, −56.16 dB, −57.90 dB, and −59.17 dB, respectively, and dominant fish species are* Coreius guichenoti* (Bleeker),* Siniperca chuatsi* (Basilewsky), and* Pelteobagrus vachelli* (Richardson). In the longitudinal direction, fish preferred to stay in some specific sections like reaches 2, 4, 7, 8, 11, and 16. Since hydrology factors change greatly in different seasons, environmental characteristics vary along the reaches, and human activities play an important role in the fish behavior, it is concluded that great cross-season changes in hydrology lead to the differences in TS and fish assemblages and that geography characteristics, especially channel geography, together with human activities influence fish longitudinal distribution. This finding provides basic knowledge of spatiotemporal distribution and assemblages of fishes in the extended reaches downstream Gezhouba Dam. In addition, it offers implications for river management. It could also serve as reference of future research on fish habitat.

## 1. Introduction

Yangtze River is the largest river in China and the third largest in the world. It originates in mount Tunggula and flows into the East China Sea, with the total length of about 6300 kilometers. The complex geological environment and climate conditions bring Yangtze River a high biodiversity [1]. There are more than 360 species of freshwater fishes in the Yangtze River. This river exhibits a seasonal flow. And water temperature is high in the summer and low in the winter. Other ecological environments also vary from season to season. Gezhouba Dam ( lowermost dam in the Yangtze River), part of the Three Georges water conservancy project, was the first dam in the Yangtze main stream and located in Yichang city, Hubei province, China, about 40 kilometers away downstream Three Georges Dam.

With the development of the acoustic methods, hydroacoustic technology has been used in fisheries research successfully for decades. It was often used to estimate fish abundance and distribution and observe fish behaviors like swimming speed or direction not only in the marine but also in the river. This technology provides a convenient and direct means to observe fish in situ without disturbance or damage of various aquatic systems [2], and it has been successfully used to monitor the fish in river both vertically [3, 4] and horizontally [5]. Horizontal sonar is usually used in shallow waters to study the fish size, migration, and abundance in the upper layer waters [6–8], and the “dead-zone” of layer waters was detected [9], while vertical sonar has been well applied to understanding of fish distribution and density in various waters including seawater and freshwater [2, 4]. Vertical sonar is also widely used in Chinese rivers [10–12]. Hydroacoustic technique has many advantages over traditional methods in studying fish size, abundance, and swimming speed, and it reduces manpower and reflects the fish behavior in natural state and is also less environment-dependent.

There existed more than 90 fish species in downstream section of the Gezhouba Dam in 1980s at the beginning of its construction. Among these species, there are some endangered and protected species at China national animal protection level, such as Chinese sturgeon (*Acipenser sinensis* Gray, CR, in the IUCN Red list) and Dabry's sturgeon. However, investigations on the catches in last decades showed that the reduction trend in fish species and fishery resources has become apparent in this downstream section [13, 14]. Since the construction of Three Gorges Dam, spatiotemporal distribution of dominant species was influenced by the changes in hydrological conditions [15]. Therefore, protection of fishery resources is of great significance, and the knowledge of the fish distribution and behavior is essential for river management.

Many studies of fish size and density in the downstream area of the Gezhouba Dam were carried out. However, almost all of them focused only on the area near the dam and the limited species like Chinese sturgeon (*Acipenser sinensis* Gray) [12, 15, 16], and the fish distribution in extended section remains unknown.

In this present study, acoustic detections coupled with fish sampling and the other datum collected were performed and this study is aimed at (1) understanding the fish distribution in the extended river section in downstream reach of Gezhouba Dam and its differences in different seasons, (2) finding out relations between the fish behaviors and environmental factors, (3) providing valuable information for fishery management and fish potential habitat.

## 2. Materials and Methods

### 2.1. Study Area

**Figure 1:**
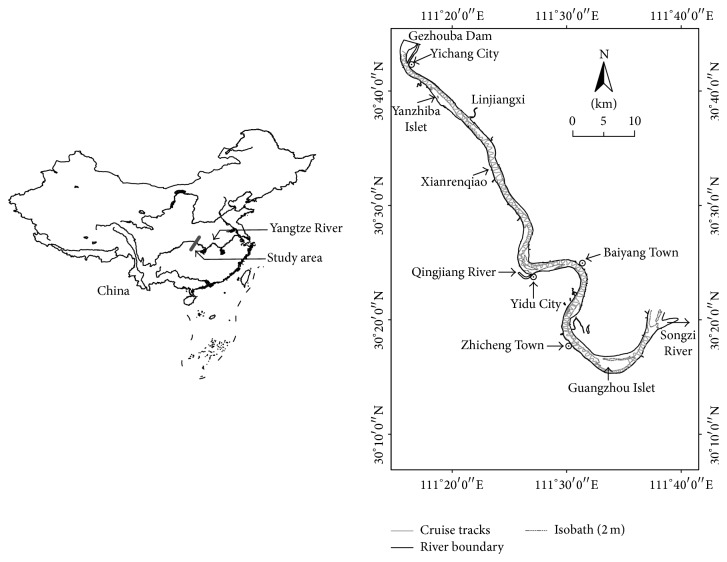
Study area, about 85 km in downstream reach of Gezhouba Dam, Yangtze River.

The study area covers the protected river reaches from downstream Gezhouba Dam (rkm 1678 km) to Songzi River (rkm 1598), with the span of about 80 km (Yangtze River estuary was defined as river kilometer (rkm) 0). The geomorphology of this area changes apparently from mountainous to flat and its hydrological characteristics are regulated by Gezhouba Dam and Three Georges Dam. The average annual flow discharge and water level at Yichang Hydrological Monitoring Station (YHMS) from 1950 to 2000, respectively, were 13900 m^3^/s and 43.8 m [17]. In the study area, there are two tributaries, one is Qingjiang River which flows into the Yangtze River, and the other named Songzi River flows out. The reaches are microbend straight from Gezhouba Dam (rkm 1768) to Yidu city (rkm 1633) and tortuous in the last 35 km (Figure 1). The geographical advantages (river flows through the whole Yichang city and the study area is located at the junction of the upper and middle Yangtze River) and economic development in this area result in frequent human activities like shipping, wading engineering, and sewage disposal, which brings about tremendous pressure to the protection of aquatic environment in this area.

### 2.2. Hydroacoustic Detections

Five acoustic detections were performed in different seasons from 2015 to 2016, using a fiberglass-reinforced plastic boat with the length 6.3 m and engine power 85 hp, respectively. The echo sounder was equipped with a 199 kHz BioSonics DT-X with a 6.7° split-beam transducer and set to a source level of 221.0 dB re 1 *μ*Pa at 1 m and a receiver sensitivity of −51.3 dB re 1 *μ*Pa. While detecting, the pulse duration was 0.4 ms and pulse rate was 5 pings/s.

The transducer was anchored on the right side of the boat and at a depth 0.5 m into the water vertically so as to sample the entire water column from 1.5 m below the water surface to 0.5 m above the bottom. The vertical motion detection was performed at a speed of about 8–10 km/h with zigzag transects using a GPS receiver (JRC, Tokyo, Japan). It took 4 days to complete one detection due to the limited longitudinal detection distance of approximately 25 km one day. All the detections were performed in the daytime from 9:00 am to 17:00 pm (waterway transports make the detection in the night dangerous).

### 2.3. Fish Sampling

Fishing with various gillnets (6, 8 cm) was carried out to obtain catches and fish species. Investigations on catches were conducted in July, November, December 2015, and January 2016. Two different habitat types were selected to investigate catches and these two habitats were classified into running water area with fast flows and pool water area with smooth water surface. In the running water area, drift nets were used to fish, while in the pool water area, set gill nets were used. Body length (in millimeter) and weight (in grams) of each fish were measured. The fish sampling area covered the 20 km downstream reach from Gezhouba Dam.

### 2.4. River Environment

In river environment description, the waterway kilometrage was divided according to channel chart supplied by the Yangtze River navigation agencies. Frequency of the human activities was divided into two categories, frequent or less. Frequent category was classified into two main types: wading engineering (type I) and anchorage zone (type II), and less frequent category refers to nonwading engineering and nonanchorage zone as previously mentioned (Table 1).

**Figure 2:**
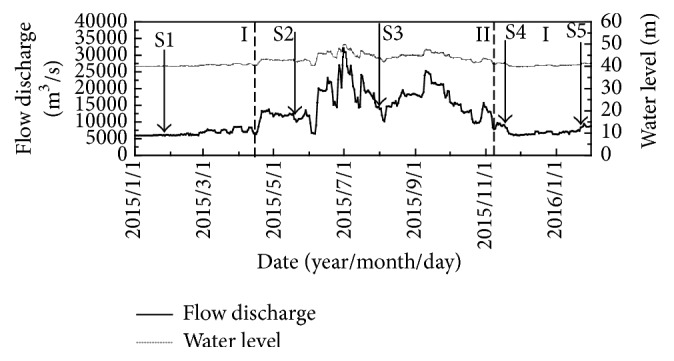
Flow discharge and water level in study area, 85 km downstream Gezhouba Dam, Yangtze River, 2015-2016.

The data of water level and flow discharge were obtained from China Three Gorges Corporation. The detection date was divided into two periods, wet seasons (area I) and flood seasons (area II) (Figure 2).

### 2.5. Acoustic and Fish Density Analysis

Acoustic data were processed by Echoview software v. 4.9 (Myriax Pty Ltd, Hobart, TAS, Australia) with times-varied gain (TVG) of 20log⁡*R*. However, not all the acoustic data were chosen. Data from water surface to 1 m below surface was filtered through a straight line due to the movement of vessels and the existence of “dead-zone.” In the same way, data of 0.5 m above the river bottom were also removed. Only the data of 1 m under the water surface and 0.5 m above river bottom were retained to be analyzed. Some noise echoes were manually identified and deleted. In the single-echo detection (SED) echogram, the maximum one-way gain compensation was set to 10 dB with TS threshold being −65 dB so as to exclude the echoes of fish below threshold value. When 4 single echoes were detected from one target with a maximum gap of 2 pings, one acceptable fish track detection (FTD) was obtained. Then information of every individual fish, such as TS, depth in water, location, and so on, was exported. The total length of every individual fish was usually calculated by a typical version equation: TS = 19.1 × log (TL) − 0.9 × log (Frequency) − 62.0 (Love, 1971) [18].

Fish density algorithm was calculated as follows:

(1) σ−bs=10TS/10 m2ρvS=Svσ−bs m−3.

${\overset{-}{\sigma }}_{bs}$ is the mean backscattering cross-section of all species (m^2^), *ρ*_*vS*_ is the volumetric fish density in the region (fish/m^3^), and *S*_*v*_ is the linear mean *S*_*V*_ value for the region (m^2^/m^3^).

## 3. Results

### 3.1. Assemblages of Fishes

Totally, 5687 fishes collected belong to 4 orders, 11 families, 38 genera, and 53 species. Of these 53 species, 13 species whose percentage (% N), respectively, exceeded 1% of the total number of catches (5687) together account for 92.84% of the total number and 87.43% of the total weight of all catches. The breeding seasons of these 13 species are all in flood seasons about from March to August. 5 species are peculiar to the upper reaches of the Yangtze River whose stays are related to the lock and opening and flow discharge of Three Gorges Dam and Gezhouba Dam (Table 2).

### 3.2. Fish Vertical and TS Distribution

**Figure 3:**
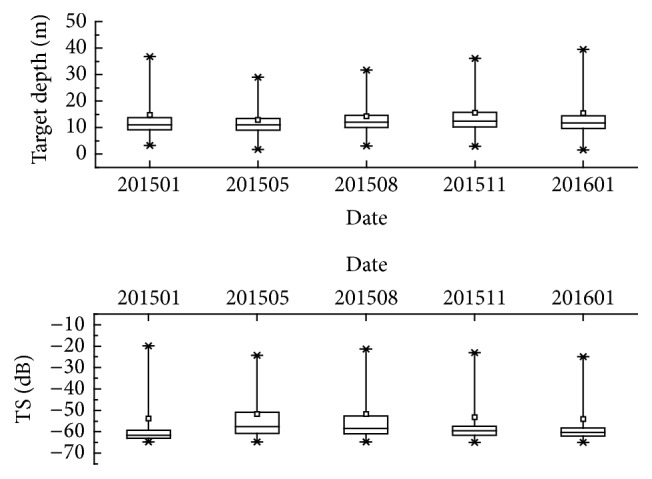
Fish vertical and TS distribution of the five hydroacoustic detections.

TS and TD (target depth) were obtained by Echoview analysis. Mean TS of the five detections were −59.98 dB, −54.70 dB, −56.16 dB, −57.90 dB, and −59.17 dB, respectively, and the mean target depth was 12.06 m, 11.47 m, 12.78 m, 13.72 m, and 12.44 m, respectively. Five acoustic detections indicated that differences of main TS distribution in various months were more significant than that of TD. The results of TS detection, respectively, in January 2015 November 2015, and January 2016 mainly ranged from −63 dB to −58 dB, while TS detection in May and July 2015 mainly ranged from −58 dB to −52 dB. In other words, target length converted from D1, D4, and D5 was bigger than that from D2 and D3 after using Love's TS-TL equation. Signals of TS > −30 dB were founded in each of five detections and they were maybe sent back by Chinese sturgeon (*Acipenser sinensis* Gray) [12]. The results of five TD detections concentrated in the depth layer from 5 to 15 m and no obvious differences were found between these detections (Figure 3).

### 3.3. Fish Longitudinal Distribution

Fish longitudinal distribution was displayed by the fish density (ind/1000 m^3^). Eighty kilometers of distance from the Gezhouba Dam to Songzi River was divided into 5 km interval reaches according to the river geomorphology and there were 16 reaches in total. Shades of the color represented the area density. Black area represented high density and white represented low density.

**Figure 4:**
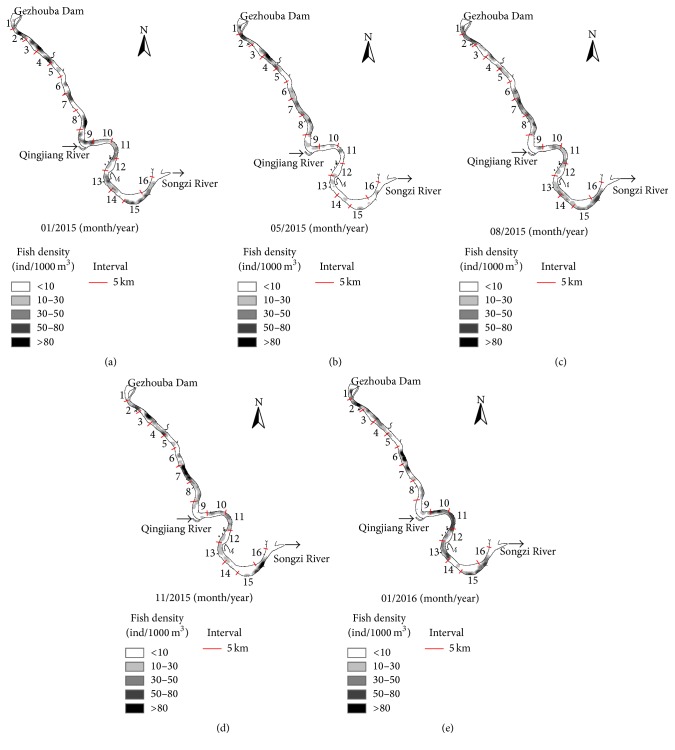
Fish longitudinal distribution of the five hydroacoustic detections. (a) Hydroacoustic detection in January 2015; (b) hydroacoustic detection in May 2015; (c) hydroacoustic detection in August 2015; (d) hydroacoustic detection in September 2015; (e) hydroacoustic detection in January 2016.

The fish gathering places were different in the five acoustic detections. Fish longitudinal distribution differed greatly between the flood and wet season. B (the hydroacoustic detection in May 2015) found that fishes mainly were distributed in the first eight reaches, while C (the hydroacoustic detection in August 2015) found more even distribution of fishes. Few fishes were distributed in reach 9 and reach 14 with anchorage zone there, and fish preferred to stay in some specific reaches like reaches 2, 4, 7, 8 11, and 16 (Figure 4).

## 4. Discussion

Most of the studies by using hydroacoustic technology focused on fish biomass, size, and distribution in large estuaries, lakes, and rivers [3, 19, 20]. Few studies reported the research findings in the longer downstream river section of a dam. This study indicated that the combination of hydroacoustic technology and traditional fish sampling turned out to be an effective way to understand the fish spatiotemporal distribution and assemblages downstream Gezhouba Dam in different seasons. The results showed significant differences in fish longitudinal distribution and size in different seasons. Fish preferred to stay in the specific reaches where river channel was curving, where there were geographical riffles and less human activities. There may be three reasons for the results. First, great changes in hydrology in different seasons may result in the different fish longitudinal distribution, assemblages, and size. Second, the geography characteristics of river channel in this study area vary along the reaches, which may influence fish gathering. Third, human activity is also a limiting factor.

Fish distribution and abundance in rivers displayed longitudinal zonation from upstream to downstream [20, 21]. Water level changes from May to October in the Yangtze River, and many fishes migrate when water level changes seasonally in the Yangtze River. In flood seasons, hydrologic factors like water temperature, depth, flow velocity, flow discharge, and nutrients change rapidly. High flow discharge could bring high flow velocity and more nutrients, which play an important role in fish behaviors including migration, feeding, and reproduction. In addition, flood seasons are fish breeding periods for most of the species in this study area. Riverine fish assemblages change rapidly or gradually with rapid or gradual changes in the physicochemical habitat [22]. Therefore, great differences in mean TS and fish longitudinal distribution between flood and wet seasons were found in this study.

Fish habitat is an essential component of ecosystem [23], while studies of fish habitats downstream Gezhouba Dam were merely limited to the habitats of large endangered individual Chinese sturgeon (*Acipenser sinensis* Gray) [24–27]. This study covers different reaches with different geography characteristics that are habitats of various fish species. Reaches 4 and 7 of this study area are straight geomorphologically with riffles where fish preferred to inhabit. Ranges of velocity and depth around the island are wider than those in other straight river sections without islands [28]. Reaches with riffles represent more complex environment beneficial for fish to avoid interference and predators. There also exists anchorage zone in reaches 4 and 7, and human waste such as deserted food and sanitary waste are discharged directly into the river here, which provides much more food and nutrients for these two reaches and make them an ideal habitat place for fish to feed and avoid disadvantage behaviors. Reaches 2, 8, 11, and 16 are curing section with mountains and riffles and with less human activities. Therefore, the environment of these reaches is much more natural and less disturbed externally. This may explain why fish preferred to stay in these reaches.

There are still some limitations in this study. Because of the fishing ban seasons (from March to May every year before 2015 and February to May from 2016), fish sampling and hydroacoustic detections could not be conducted simultaneously. The calculation results of the corresponding fish length of five acoustic detections were 1.64 cm, 3.09 cm, 2.59 cm, 2.10 cm, and 1.80 cm, respectively, by using the typical version equation: TS = 19.1 × log (TL) − 0.9 × log (Frequency) − 62.0 [18]. However, these calculation results significantly differ from actual measurement results of fish length of the catches. Since few researches on conversion equations of TS values and fish length about fish species in Yangtze River were reported, there have been no appropriate equations to match these two results so far. According to the average annual flow discharge and water level at YHMS in the last 50 years (from 1950 to 2000) and the situation of hydroacoustic detection in recent years [12, 17], the periods of wet seasons and flood seasons are almost the same every year. They could be divided into flood seasons and wet seasons (Figure 2). Therefore, five hydroacoustic detections were conducted in these two seasons: three in the wet seasons and two in the flood seasons. What is more, high flow discharge (when flow discharge > 20000 m^3^/s) in the flood seasons brings high velocity and much more bubbles which make the hydroacoustic detection unsafe and inaccurate. Therefore five hydroacoustic detections were conducted in the two periods (Figure 2, wet seasons and flood seasons).

Previous studies focused on the distribution and spawning grounds of Chinese sturgeon [16, 24, 29] or fish assemblages and behaviors in the downstream zone adjacent to Gezhouba Dam. This research extended the study area to 80 km away from Gezhouba Dam to offer the knowledge of fish longitudinal distribution and size in different seasons. Our study results demonstrated that fish distribution was influenced by both environment factors and human activities. Fish habitat loss has become a big threat to aquatic biodiversity [1]. Although quantitative factors like velocity, dissolved oxygen, and fish assemblages are not measured, the reaches of Yangtze River reported by this study where fish preferred to inhabit could be considered as potential fish habitats, which would be helpful for future research on fish habitat. Furthermore, how Gezhouba Dam affects the fish is still unknown, but the effects of Gezhouba Dam on fish survival were exemplified by the Acipenseriformes [30, 31]. Researches on the effects of Gezhouba Dam and conservations for fish biodiversity are also essential in the future work.

Based on this study findings, the suggestions for river management are as follows.

1. Fishing ban seasons may be adjusted to containing the whole flooding seasons especially July and August since the breeding seasons of dominant fish species such as* Coreius guichenoti* (Bleeker),* Siniperca chuatsi* (Basilewsky), and* Pelteobagrus vachelli* (Richardson) in this study area are in the flooding seasons.
2. Strengthen the supervision over poaching and overfishing in the specific reaches mentioned in this study where fishes prefer to inhabit.
3. Select wading engineering location scientifically to avoid the loss of fish habitat.

## Figures and Tables

**Table 1:** Parameters of the divided reaches.

Reach ID	Left rank	Right rank	Human activity	Channel geography
1	Revetment	Revetment	Less	Curing
2	Revetment	Mountain	Less	Curing
3	Revetment	Revetment	Less	Straight
4	Revetment	Mountain and riffle	Frequent (II)	Straight
5	Revetment	Revetment	Less	Straight
6	Revetment	Revetment	Less	Straight
7	Revetment and riffle	Revetment and riffle	Frequent (II)	Straight
8	Mountain	Revetment and riffle	Less	Curing
9	Revetment and riffle	Revetment and riffle	Frequent (I)	Straight
10	Revetment	Revetment and riffle	Less	Straight
11	Mountain	Revetment and riffle	Less	Curing
12	Revetment	Revetment and riffle	Less	Straight
13	Revetment	Revetment	Less	Straight
14	Revetment	Revetment	Frequent (I)	Straight
15	Revetment and riffle	Revetment	Less	Curing
16	Revetment and riffle	Mountain	Less	Curing and straight

**Table 2:** Catches catalogue downstream Gezhouba Dam, Yangtze River.

Species	Number (*N*)	% *N*	Weight (*W*)	% *W*	Mean TL
*Coreius guichenoti* (Bleeker)	1665	29.28	363385.10	36.88	275
*Siniperca chuatsi* (Basilewsky)	1492	26.24	199257.10	20.22	156
*Pelteobagrus vachelli* (Richardson)	642	11.29	51837.86	5.26	154
*Rhinogobio cylindricus* Günther^*∗*^	330	5.80	25424.00	2.58	201
*Parabramis pekinensis* (Basilewsky)	237	4.17	80081.90	8.13	283
*Siniperca scherzeri* Steindachner	188	3.31	36476.70	3.70	214
*Rhinogobio typus* Bleeker	145	2.55	20825.20	2.11	244
*Xenocypris davidi* Bleeker	130	2.29	11253.90	1.14	196
*Mystus macropterus* (Bleeker)	113	1.99	19892.90	2.02	278
*Xenocypris davidi* Bleeker	108	1.90	12287.60	1.25	146
*Leiocassis crassilabris* Günther	95	1.67	6657.00	0.68	138
*Carassius auratus* (Linnaeus)	77	1.35	21265.30	2.16	167
*Leptobotia elongata* (Bleeker)^*∗*^	58	1.02	12850.30	1.30	258
*Cyprinus carpio* Linnaeus	52	0.91	40802.30	4.14	169
*Pseudolaubuca sinensis* Bleeker	49	0.86	2858.80	0.29	173
*Saurogobio dabryi* Bleeker	42	0.74	2664.00	0.27	183
*Pelteobagrus fulvidraco* (Richardson)	35	0.62	2601.00	0.26	162
*Silurus asotus* Linnaeus	27	0.47	15022.70	1.52	281
*Squalidus argentatus* (Sauvage et Dabry)	24	0.42	678.60	0.07	122
*Hemibarbus maculatus* Bleeker	18	0.32	2689.80	0.27	187
*Pseudobrama simoni* (Bleeker)	16	0.28	658.90	0.07	145
*Pelteobagrus nitidus* (Sauvage et Dabry)	14	0.25	309.50	0.03	128
*Leiocassis longirostris* Günther	13	0.23	4842.30	0.49	307
*Hypophthalmichthys molitrix* (Cuvier et Valenciennes)	11	0.19	2320.80	0.24	212
*Coreius guichenoti* (Sauvage et Dabry)^*∗*^	10	0.18	5494.60	0.56	360
*Squaliobarbus curriculus* (Richardson)	9	0.16	2442.50	0.25	280
*Silurus meridionalis* Chen	9	0.16	12231.90	1.24	545
*Megalobrama amblycephala* Yih	8	0.14	5533.70	0.56	371
*Pseudobagrus pratti* Günther	7	0.12	327.20	0.03	184
*Hemiculter bleekeri* Warpachowsky	6	0.11	103.40	0.01	128
*Pseudobagrus truncates* (Regan)	6	0.11	102.00	0.01	117
*Hemiculter leucisclus* (Basilewsky)	5	0.09	204.90	0.02	123
*Ctenopharyngodon idellus* (Cuvier et Valenciennes)	5	0.09	13477.81	1.37	606
*Tinca tinca* (Linnaeus)	5	0.09	1690.90	0.17	284
*Culter alburnus* Basilewsky	5	0.09	2185.60	0.22	167
*Odontobutis obscura* (Temminck & Schlegel)	3	0.05	34.40	<0.01	10
*Hyporhamphus intermedius *(Cantor)	3	0.05	317.90	0.03	201
*Jinshaia sinensis* (Sauvage et Dabry)^*∗*^	3	0.05	147.50	0.01	101
*Parabotia fasciata* Dabry	2	0.04	82.40	0.01	188
*Lepturichthys fimbriata* (Günther)	2	0.04	11.80	<0.01	116
*Culter molitorella* (Cuvier et Valenciennes)	2	0.04	170.70	0.02	219
*Culter mongolicus mongolicus* (Basilewsky)	2	0.04	1090.50	0.11	375
*Culter oxycephaloides* Kreyenberg et Pappenheim	2	0.04	1016.10	0.10	431
*Myxocy prinus asiaticus* (Bleeker)	2	0.04	235.40	0.02	183
*Pelteobagrus eupogon* (Boulenger)	2	0.04	110.00	0.01	19
*Saurogobio gymnocheilus *Lo Yao & Chen	1	0.02	67.60	0.01	224
*Opsariichthys bidens* Günther	1	0.02	32.00	<0.01	15
*Misgurnus anguillicaudatus* (Cantor)	1	0.02	37.00	<0.01	162
*Channa argus* (Cantor)	1	0.02	781.50	0.08	432
*Gobiobotia filifer* (Garman)	1	0.02	7.50	<0.01	107
*Aristichthys nobilis* (Richardson)	1	0.02	59.40	0.01	180
*Rhinogobio ventralis* Sauvage et Dabry^*∗*^	1	0.02	179.60	0.02	251
*Spinibarbus sinensis* (Bleeker)	1	0.02	224.10	0.02	267
Total	5687	100	985343.47	100	

Number (*N*), percentage number (%  *N*), weight (in grams, *W*), percent weight (%  *W*), and mean total length (mm, mean TL). *∗* means that this species is peculiar to the upper reaches of the Yangtze River.
